# Multiplicity Eludes Peer Review: The Case of COVID-19 Research

**DOI:** 10.3390/ijerph18179304

**Published:** 2021-09-03

**Authors:** Oliver Gutiérrez-Hernández, Luis Ventura García

**Affiliations:** 1 Department of Geography, University of Málaga, 29010 Málaga, Spain; 2 Institute of Natural Resources and Agrobiology of Seville (IRNAS), Spanish National Research Council (CSIC), 41012 Seville, Spain; lv.garcia@csic.es

**Keywords:** multiple hypotheses testing, multiple testing problem, false discovery rate (FDR), environmental research, epidemiology, health geography, SARS-CoV-2

## Abstract

Multiplicity arises when data analysis involves multiple simultaneous inferences, increasing the chance of spurious findings. It is a widespread problem frequently ignored by researchers. In this paper, we perform an exploratory analysis of the Web of Science database for COVID-19 observational studies. We examined 100 top-cited COVID-19 peer-reviewed articles based on *p*-values, including up to 7100 simultaneous tests, with 50% including >34 tests, and 20% > 100 tests. We found that the larger the number of tests performed, the larger the number of significant results (r = 0.87, *p* < 10^−6^). The number of *p*-values in the abstracts was not related to the number of *p*-values in the papers. However, the highly significant results (*p* < 0.001) in the abstracts were strongly correlated (r = 0.61, *p* < 10^−6^) with the number of *p* < 0.001 significances in the papers. Furthermore, the abstracts included a higher proportion of significant results (0.91 vs. 0.50), and 80% reported only significant results. Only one reviewed paper addressed multiplicity-induced type I error inflation, pointing to potentially spurious results bypassing the peer-review process. We conclude the need to pay special attention to the increased chance of false discoveries in observational studies, including non-replicated striking discoveries with a potentially large social impact. We propose some easy-to-implement measures to assess and limit the effects of multiplicity.

## 1. Introduction

Multiplicity is a common problem in environmental and epidemiological research [1,2]. It is especially critical in observational studies in which many questions (that is, many individual tests) are asked on many variables measured in the same data set [3]. When the results of multiple testing are evaluated individually (i.e., on a per-test basis), the chance of obtaining false positives (“spurious discoveries”) increases with the number of simultaneous tests performed. For example, on a table of 10 tests, the probability of obtaining at least one spurious discovery may increase to 40%, instead of the 5% declared as the per-test level of significance, and performing 50 simultaneous tests almost ensures (90%) at least one false discovery. In recent decades, most researchers, referees, and editors have ignored or diminished the problem [4].

The COVID-19 pandemic has multiplied observational research by looking for ways to fight the pandemic as best and as quickly as possible [5]. Although research advances related to COVID-19 have been impressive over the last year, the mountain of false positives probably continues to grow; it results in the generation of inconsistent evidence that complicates the development of proper public health guidelines [6].

This paper deals with the crucial role of the “multiplicity problem” when analyzing data from observational studies in which many research questions are tested. It is especially critical when many tests are performed looking for statistically significant effects without clearly specifying a prior hypothesis. Multiple testing is usually followed by insufficiently documented selective inference (e.g., reporting or discussing only the statistically significant relationships on a per-test basis while ignoring the total number of inferences involved). It can lead to reader confusion about the reported discoveries’ actual relevance (and potential replicability) [7].

We first perform a preliminary analysis of COVID-19 peer-reviewed scientific literature that uses *p*-values as a primary tool to determine the potential significance of their results. We are particularly interested in evaluating how many *p*-value-based articles involving multiple testing address multiplicity or, at least, mention it as a potential limitation.

We hypothesize that a relevant proportion of COVID-19 peer-reviewed studies involving substantial levels of multiplicity largely ignore its potential effects on their conclusions. Additionally, we revisit a recently published case study on COVID-19 [8], including a striking discovery, reported widely in the media. Although we have already mentioned different potential methodological flaws of that work in a recently published correspondence article [9], we will analyze it in greater depth here, only regarding the multiplicity problem. It clearly illustrates the potential consequences of ignoring multiplicity in exploratory studies and the importance of addressing and informing readers to avoid mere suggestions from being presented as solid peer-reviewed evidence to the public and authorities.

## 2. Methods

### 2.1. Background

The *p*-value approach to hypothesis testing involves comparing the probability of observing the calculated test statistics under the null hypothesis (*p*-value), with a fixed threshold alpha, usually 0.05 or 0.01. The result is significant (i.e., the null hypothesis is rejected) if the obtained *p*-value is lower than (or equal to) alpha. Otherwise, the null hypothesis is not rejected, and the result is considered “non-significant”. This frequentist approach has been used for the last century, mainly since R. A. Fisher provided the means to calculate the *p*-value in a wide variety of situations [10].

When the required assumptions of the statistical procedure used are met, the probability of obtaining at least one significant spurious result (that is, rejecting the null hypothesis being true, type I error) is controlled at the fixed threshold. However, this is not true when several or many (n) simultaneous tests are carried out since, as mentioned above, the overall type I error can overgrow up to 40 (n = 10) or 90% (n = 50). It is, therefore, necessary to apply specific procedures to control for type I error inflation when performing multiple tests.

There are two main conceptual approaches to handle multiple testing. On the one hand, controlling the family-wise error rate (FWER) extends the aim of single tests to a family of several (or many) tests: control the probability of even one false positive in the entire set at the level alpha. It is suitable when it is crucial to avoid any false positive, and the number of simultaneous tests is low—on the other hand, controlling the false discovery rate (FDR), i.e., the expected proportion of false discoveries [11]. It is a more powerful method that allows for handling tables with hundreds or thousands of simultaneous tests. Its use is recommended when it is more important to control the expected proportion of false discoveries than the probability of having even one false positive (see [3] for further details).

Many previous studies have addressed the multiplicity issue, called attention to its potential consequences [3,4,12,13], and explained the fundamental theoretical issues and main calculation algorithms [14]. Hence, we will not expand the topic further.

### 2.2. Multiplicity Analysis in COVID-19 Peer-Reviewed Research

We were interested in assessing the potential extent of the uncorrected multiplicity in COVID-19 correlational research based on *p*-value inferences. For this, we explored the Web of Science Core Collection (WOS). WOS is one of the most prestigious scientific literature databases, including articles from peer-reviewed journals selected according to a set of pre-established quality and impact criteria. We searched for articles including COVID-19 or SARS-CoV-2 in their abstracts, together with explicit reference to *p*-values directly related to the significance of the results obtained. We also referenced correlation analysis in the search, since it is well-known that exploring large correlation matrices is a frequent source of type I error inflation [15]. See search details in the Supplementary Material.

After conducting the search, we retrieved 624 references, which were ranked in descending order according to their citations (used here as a proxy- of their potential influence in the scientific community). The top-100 potentially most influential articles from the set were selected for further analysis. Five review articles were discarded and replaced by the following five articles in the ranking (see supplementary material for details). We recorded the journals where the selected papers were published and classified them according to the fixed WOS knowledge categories.

For each reviewed paper, we estimated by counting the number of *p*-values and looked for some reference to multiplicity, including methods for controlling its effects or mentioning the potential resulting limitations. Specifically, for each paper, we estimated the number of inferences based on *p*-values (sometimes through non-numerical references in text) and the number of significant *p*-values (*p* < 0.05). When possible, the number of *p*-values below more demanding thresholds (e.g., 0.01 and 0.001) was also estimated. The same counts were performed for the abstract and the main text independently. We recorded the number of valid values and calculated ordinal statistics (median, quartiles) for every generated count variable. Bivariate ordinal statistics (Spearman rank coefficient) and a Chi-squared test for comparing proportions were also performed. We fixed a per-test significance level of *p* < 0.001. When necessary, the significance per-test threshold was corrected for multiplicity by applying the procedures mentioned in Section 2.3.1. Statistical analysis was performed using the R functions *corr.test()*, *prop.test()*, *p.adjust()* and *quantile()* [16].

### 2.3. A COVID-19 Case Study

As stated above, we use the paper of Rodríguez-Barranco et al. (2021) [8] as a case study. This article has had a widespread diffusion since it was first published in early view several months ago. Televisions, newspapers, and social networks around the world echoed its alarming discovery: walking a dog increased your risk of contracting COVID-19 by nearly 80% (see, for example, [17]). Even today, internet search engines will return hundreds of results to connect COVID-19 and dog walking. This article, which stands out in the PlumX metrics (mainly in mentions and social media diffusion metrics), is an excellent example of an exploratory study published in a rigorous peer-reviewed journal that includes an isolated (non-replicated) striking discovery with high social impact.

Rodríguez-Barranco et al. (2021) [8] performed an online survey to identify risk factors for contracting COVID-19. They tested differences in the estimated prevalence of COVID-19 between categories of the predictor variables studied. They concluded that only six out of the >40 used single predictors were significantly related to differences in COVID-19 prevalence. These were living with a COVID-19 patient, smoking, disinfecting purchased products upon arrival at home, using public transportation, walking with pets, and working on-site at the workplace during confinement. They also fitted a multivariate risk logistic regression model on a subset of predictors previously selected based on the significance (*p*-value) of their bivariate relationship with the dependent variable. The final fitted significant model retained five predictors (see Table 5 from Rodríguez-Barranco et al. 2021 [8]), including walking a pet. They reported that their results demonstrated that walking a pet significantly increases the risk of contracting COVID-19 by 78%.

#### 2.3.1. Multiplicity Analysis

We used different procedures belonging to the two conceptual approaches mentioned above to control for spurious findings. We used a powerful sequential method to control the family-wise error rate (FWER) [18]. Likewise, we also controlled for the false discovery rate (FDR), according to Benjamini and Hochberg (1995) [11], using the *p.adjust()* function of the R Stats Package [16]. As stated above, fitting the final logistic model involved carrying out at least 19 statistical tests. However, only the results of five judged significant were provided. As an approximation of the potential effects of this “hidden multiplicity” on model coefficient significance, we calculated the adjusted *p*-values for the finally fitted coefficients using the option for partially unknown *p*-values sets available in the function *p.adjust()* [16].

## 3. Results

### 3.1. Multiplicity in Peer-Reviewed Research on COVID-19

**Figure 1:**
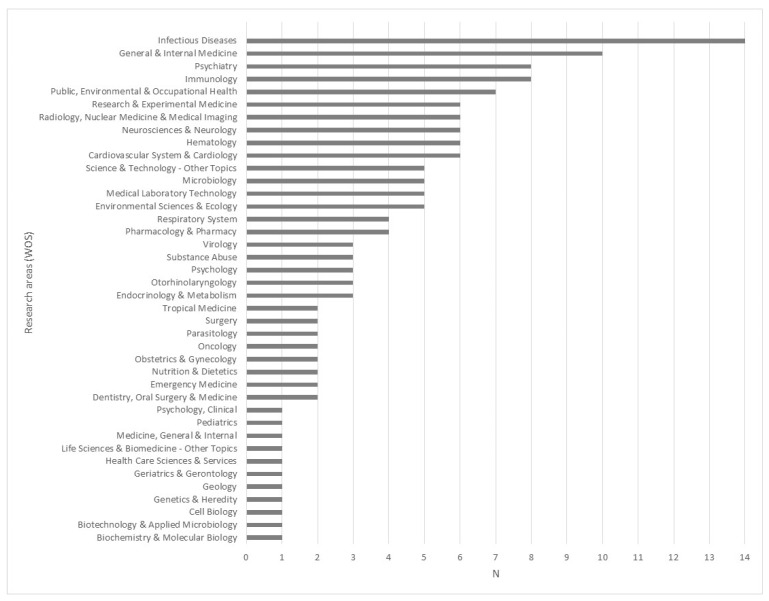
Distribution of the journals that published the 100 reviewed articles across the subject categories defined on the Web of Science Core Collection.

Supplementary material includes the basic data on the search carried out (search criteria and string, date) and search results (year, journal and paper DOI), respectively. The analyzed papers came from 86 different WOS Core Collection journals (See Supplementary Information), related to twenty different WOS knowledge categories (Figure 1). As expected, medical specialties predominate.

Half of the 100 COVID-19 papers reviewed included at least 34 *p*-values, 25% included over 76 tests (Table 1), and about 20% had over 100 tests. The corresponding values for median and upper quartile for the total number of *p*-values, <0.05, and <0.01, were 16 and 36 and 10 and 26, respectively (Table 1). We also counted the number of *p*-values < 0.001, but only for 81% of the reviewed papers, compared to 100% for *p* < 0.05 and 97% for *p* < 0.01 counts (Table 1).

Counts for the number of total, *p* < 0.05, *p* < 0.01 and *p* < 0.001 *p*-values in the abstracts of the 100 studied papers are summarized in Table 1. Values of median and quartiles for each count variable are also shown.

The numbers of *p*-values passing the most usually considered significance thresholds (i.e., *p* < 0.05, *p* < 0.01, and *p* < 0.001) counted in the main text of the analyzed papers were highly and positively correlated with each other and with the total number of tests performed. The number of *p* < 0.05 *p*-values was the one that was most related to the total number of tests carried out in the article (r = 0.87, *p* < 10—6). Similar strong correlations were found between the *p*-value counts gathered from the abstracts (Table 2).

Crossed correlations between the *p*-value counts performed in the abstracts and the corresponding *p*-values gathered from the papers’ main texts are shown in Table 2. There was a weak, non-significant relationship (Spearman r = 0.18—0.31) between the number of tests counted in the paper’s main text and any of the different *p*-count variables from the abstracts. Nevertheless, there was a strong significant (r = 0.61, *p* < 10^−6^) crossed correlation between the main text and abstract *p* < 0.001 counts. The values of the abstract-main text crossed correlations decrease to 0.41 for *p* < 0.01 counts, to 0.31 for *p* < 0.05 counts, and to 0.26 for total *p*-counts. These results suggest that counting the *p*-values below the most significant threshold available in the abstract (*p* < 0.001, if possible) gives the best idea of what is going on in the main text of the paper. Looking at the total number of *p*-values in the abstract or counting *p*-values under less significant thresholds is of scarce interest, except for the *p* < 0.01 counts (Table 2).

Finally, as it seems clear from the results in Table 2, there is a strong selective inference when presenting the final, most relevant results in the article’s abstract. In fact, the average proportion of significant (*p* < 0.05) results in the main text of the 100 analyzed papers was 0.55, against 0.91 in the abstracts of the same articles, a statistically highly significant difference between proportions (*p* < 0.0001) after performing X^2^ test). Moreover, about 80% of the reviewed abstracts included only (100%) significant results.

On the other hand, except in one case [19], none of the revised papers using large numbers of *p*-values explicitly applied any procedure to control table-wise type I error inflation. Moreover, a significant proportion (>50%) of reviewed studies conducting post hoc pair-wise group comparisons in a univariate context did not (report or) effectively correct.

### 3.2. Case Study

Table 3 summarizes the multiplicity analysis of the *p*-values resulting from the bivariate analyses performed in Rodríguez-Barranco et al. (2021) [8].

Considering the results of either the FWER or FDR approaches, it can be concluded that the only strong (significant) signal emerging from the whole explored bivariate relationships with COVID-19 presence is “living with a COVID-19 patient”. No other relationship remained significant after correcting for multiplicity effects. It can also be concluded from the results of the multivariate analysis (Table 3) that only the predictor “living with a COVID-19 patient” is significant after correcting for multiplicity.

These results suggest that the significant effect of walking the pet on COVID-19 transmission is probably a false discovery. See [9] for other methodological concerns about this case study.

## 4. Discussion

A crucial outcome of our research is that the potential effects of even extreme multiplicity on the significance of published COVID-19 results have been entirely ignored by the authors and by referees and editors of influential peer-reviewed journals.

The results of our case study suggest that, after considering the effects of multiplicity, Rodríguez-Barranco et al. (2021) [8] were very adventurous to offer a conclusion that stated: “The results of this study demonstrate that living with dogs, working on-site, purchasing essential commodities by using home delivery service, and especially living with a COVID-19 patient have been the main routes of transmission of SARS-CoV-2 during the most restrictive period of confinement in Spain”.

Many other non-replicated outstanding discoveries have been reported in observational studies [4,12,20,21]. This fact is not surprising, given that the potential effect of dozens or even thousands of simultaneous tests on the probability of having isolated spurious discoveries is largely ignored. As our results show, the larger the *p*-values set, the larger tend to be the number of usually considered significant (*p* < 0.05) results. If the problem is ignored, several or many of those significant results may be false discoveries. This particularly concerns observational studies oriented to explore relationships among many variables systematically. In those studies, the probability of getting extreme results under the null hypothesis (i.e., spurious significant results) is much higher than expected in planned research because of the effects derived from data dredging. In these circumstances, it is necessary to account for multiplicity and interpret the relevance of the discoveries considering the previous knowledge.

It is necessary to emphasize that hypothesis testing is a research tool that informs the likelihood of observing the results obtained under the null hypothesis and cannot directly reveal causes or the scientific relevance of a “statistically significant result”.

As we recently stated [6,22], causal inference is multifactorial when using observational/non-experimental evidence. A single hypothesis test result cannot be taken as evidence of anything relevant. In this sense, the classic epidemiological criteria for causality [23], established the necessity of having a temporal relationship (i.e., exposure precedes outcome), reproducibility of results across studies in different populations, a noticeable strength of association (since a weak association is more likely to be biased or confounded) and biological plausibility before one can think that a statistically significant result may be a really relevant result. Unfortunately, neither the statistical control of multiplicity nor the consideration of these classic epidemiological criteria was considered in the work that we have analyzed as a case study, which led to a probable spurious finding of high social impact.

On the other hand, our results show that the number of total *p*-values based inferences in the abstract is not very informative about the corresponding number of inferences in the paper’s main text. Instead, researchers seem to prefer to pick up and include in the abstract as most highly significant *p*-values as they can. Due to this, the abstract’s variable most significantly correlated to the corresponding one in the article’s main text is the number of *p*-values below 0.001. In summary, the abstracts give the reader an excellent picture of the number of highly significant results in the whole paper, but scarce information about the number of non-significant results or the multiplicity level in the article. Therefore, they suffer from poorly documented selective inference.

### 4.1. A Word of Caution on Spurious Findings

Throughout the COVID-19 crisis, research institutions, publishers, and scientists have done their best to enable promising proposals, advances, and achievements to reach the health authorities, the media, and society as soon as possible [24]. There have been impressive cooperative advances in many research areas and many papers, most of which provide valuable information, although not always well interpreted [25].

Never have so many people, or non-specialized journalists, used the terms significant/not significant, subjected (or not) to peer review, and frequently refer to the conclusion’s reliability, relevance, or provisional nature of the published results [26].

Unfortunately, until now, multiplicity has not benefited from these advances, and the media, public, and scientists are not interested in its potential effects on multiplying the chance appearance of striking but false discoveries. The critical consequence of those false discoveries may be the loss of science’s prestige and credibility, which it took so long to establish [27,28,29].

The constant repetition of incredible achievements that put society on guard and turn out to be spurious to do enormous damage and confuses the uncertainty inherent in science [30].

### 4.2. An Armistice in the p-Wars: Discuss Freely, but Inform about Multiplicity

In recent decades, there has been much controversy refers to the multiplicity issue [31,32]. Despite some scientist’s claim against hypothesis testing, the *p*-value is a widely used inferential statistic to test hypotheses [3,7]. Dropping *p*-value hypothesis testing because of its limitations is unlikely [33]. Nevertheless, interpreting the *p*-value requires a proper context [34], and understanding the limitations and variability of *p*-values is also crucial for interpreting results correctly [35].

Our preliminary results have shown that, even in studies with high levels of multiplicity, the authors ignore its potential effects on the “highly significant” discoveries they made.

Abstracts from the reviewed 100 papers included a median of three significant *p*-values accompanying the most relevant results of the paper. Without information on the multiplicity degree of the entire study, the reader would be oblivious to whether those *p*-values would remain significant after correcting for alpha inflation.

For this reason, the authors of observational studies with unexpected striking results should be required to report the level of multiplicity involved and discuss, in the study limitations section, what consequences it could have on interpreting the results of multiple tests *p*-values, and also on the convenience (or not) of applying procedures to control the rate of false discoveries. On the other hand, providing full *p*-values to allow for external evaluation of multiplicity and a detailed description of the procedures used that may involve hidden multiplicity (for example, selection of predictors for model building [9,13]) should be mandatory. It does not hurt the authors to discuss the results based on unadjusted *p*-values, but in a transparent way and without ignoring the context of multiplicity in which they do it.

## 5. Conclusions

Multiplicity is a common issue in observational studies. Our results have shown that of the 100 COVID-related papers reviewed here, 50% included over 34 simultaneous tests, with 20% including over 100 tests. We found that the higher the total number of tests performed in the paper, the higher the number of significant results obtained. Abstracts suffered from poorly documented selective inference favoring the highly significant results, with scarce or null information about the non-significant results or the multiplicity level in the article. About 80% of the abstracts included only significant *p*-values.

Only one of the 100 papers addressed the inflation error induced by multiplicity, leading to a highly likely inclusion of spurious results that bypass the peer-review process. As shown here, being oblivious to the multiplicity issue can cause potentially spurious findings, making the headline an issue that is further fostered if the findings are striking and media attractive. We argue that authors and reviewers of observational studies involving multiple testing, especially those with an enormous social impact, should pay special attention to the increased chance of false positives derived from the multiplicity effect.

Some easy-to-implement measures that would help to assess multiplicity could be: (1) provide full numeric *p*-values instead of prompts against a set threshold. (i.e., *p* < 0.0 …); (2) report the total number of tests performed; (3) offer a detailed description of the procedures used that may involve multiplicity (for example, selection of predictors for model building); (4) in the study limitations section, discuss the potential effect of multiplicity on the main discoveries reported in the abstract. Moreover, the suitability (or not) of applying some procedure to control the rate of spurious findings should be explicitly considered.

## Figures and Tables

**Table 1:** Quartiles (Q25, Q50, Q75) of the distribution of the number of *p*-values per paper estimated by counting in 100 peer-revised top papers recovered from the Web of Science Core Collection database (see methods section). Valid data (*N*), and ordinal statistics are provided for both counts of total (“*p*-values”) and significant (“*p*-values < x”, x = 0.05, 0.01, 0.001) *p*-values. Counts corresponding to the main text and abstracts are presented separately.

	*N*	Median	Q25	Q75
**Main text**				
*p*-values	100	34	12	76
*p*-values < 0.05	100	16	6	36
*p*-values < 0.01	97	10	4	26
*p*-values < 0.001	81	6	2	17
**Abstract**				
*p*-values	100	4	2	6
*p*-values < 0.05	100	3	2	6
*p*-values < 0.01	87	2	1	3
*p*-values < 0.001	78	2	0	3

**Table 2:** Spearman correlation coefficients between count variables gathered from the main text (T), and abstract (A) of the 100 analyzed peer-reviewed papers. Numbers following the capital letters (T or A) refer to the different *p*-value thresholds considered by performing the counting (0.05, 0.01, 0.001). *p*-values passing the FDR-corrected threshold value for a marginal threshold *p* = 0.001 are in italics. Correlations exceeding 0.50 are in bold.

	TP	T005	T001	T0001	AP	A005	A001
**T005**	***0.87***						
**T001**	***0.81***	***0.96***					
**T0001**	***0.67***	***0.83***	***0.90***				
**AP**	0.26	0.27	0.27	0.32			
**A005**	0.31	0.38	0.39	*0.44*	***0.93***		
**A001**	0.20	0.34	*0.41*	*0.49*	***0.73***	***0.81***	
**A0001**	0.18	0.30	*0.41*	***0.61***	***0.58***	***0.67***	***0.85***

**Table 3:** Multiplicity analysis of the bivariate and multivariate results in Tables 1 to 5 from Rodríguez-Barranco et al. (2021) [8]. Unadjusted *p*-values correspond to the original results. Adjusted *p*-values were obtained after controlling for the family-wise error rate (FWER, type I error) or false discovery rate (FDR, expected proportion of false rejections to total rejections) at the 0.05 level. Two different correction methods were considered: the sequential Holm (1979) procedure (labeled as FWER in the table) and the Benjamini and Hochberg (1995) [11] procedure (labeled as FDR in the table). Significant *p*-values, according to the different criteria, are enhanced in bold. *p*-value adjustments were performed using the *p.adjust* R function [16].

Bivariate Analysis	Unadjusted	Adjusted *p*-Values	
	*p*-Values	FWER	FDR
Live with a COVID-19 patient	0.001	0.043	0.043
Smoke	0.003	0.126	0.057
Product disinfection	0.004	0.164	0.057
Use of public transportation	0.007	0.280	0.075
Walk the pet	0.024	0.936	0.206
Work on site	0.030	1.000	0.215
**Multivariate analysis**			
Living with a COVID-19 patient	<0.001	0.009	0.009
Disinfection of food products	0.009	0.171	0.162
Traveling to the workplace	0.028	0.532	0.476
Pet walk	0.037	0.703	0.592
Food purchase modality	0.056	1.000	0.840

## Data Availability

The complete data set that supports the findings of this study is available from the corresponding author, O.G.H, upon reasonable request.

## Supplementary Materials

The following are available online at https://www.mdpi.com/article/10.3390/ijerph18179304/s1, S1: Search details and results.
