# Supplementary material for: Multiplicity Eludes Peer Review: The Case of COVID-19 Research
Source: Int J Environ Res Public Health. 2021 Sep 3;18(17):9304. doi: 10.3390/ijerph18179304 (PMC8430657; doi:10.3390/ijerph18179304)
Supplement: Supplementary file 1 [file ijerph-18-09304-s001.zip › ijerph-1329598-supplementry.pdf]

S1 - SUPPLEMENTARY INFORMATION

SEARCH DETAILS

DATE 2021/04/10
FILTER ARTICLES, ENGLISH, WOS CORE COLLECTION
STRING AB=((COVID-19 OR SARS-COV-2) AND SIGNIFICA* AND CORRELAT* AND P*.0)

SEARCH RESULTS

TOP 100 out of 624 recovered records, ranked by citations. Records in italics were excluded from analysis

Rank	Year	Source	DOI
**1**	2020	JOURNAL OF TRAVEL MEDICINE	10.1093/jtm/taaa037
**2**	2020	THROMBOSIS AND HAEMOSTASIS	10.1055/s-0040-1710018
**3**	2020	INVESTIGATIVE RADIOLOGY	10.1097/RLI.0000000000000670
**4**	2020	BRITISH JOURNAL OF HAEMATOLOGY	10.1111/bjh.16659
**5**	2020	INVESTIGATIVE RADIOLOGY	10.1097/RLI.0000000000000674
**6**	2020	MEDECINE ET MALADIES INFECTIEUSES	10.1016/j.medmal.2020.03.007
**7**	2020	LANCET HAEMATOLOGY	10.1016/S2352-3026(20)30216-7
**8**	2020	EMERGING MICROBES & INFECTIONS	10.1080/22221751.2020.1770129
**9**	2020	INTERNATIONAL JOURNAL OF MENTAL HEALTH AND ADDICTION	10.1007/s11469-020-00289-x
10	*2020*	POLISH ARCHIVES OF INTERNAL MEDICINE-POLSKIE ARCHIWUM MEDYCYNY WEWNETRZNEJ	10.20452/pamw.15272
**11**	2020	CLINICA CHIMICA ACTA	10.1016/j.cca.2020.06.026
**12**	2020	VIRAL IMMUNOLOGY	10.1089/vim.2020.0062
**13**	2020	INTERNATIONAL FORUM OF ALLERGY & RHINOLOGY	10.1002/alr.22580
**14**	2020	INFECTION	10.1007/s15010-020-01427-2
**15**	2020	JOURNAL OF CLINICAL MICROBIOLOGY	10.1128/JCM.02107-20
**16**	2020	STROKE	10.1161/STROKEAHA.120.030373
**17**	2020	INTERNATIONAL JOURNAL OF GYNECOLOGY & OBSTETRICS	10.1002/ijgo.13165
**18**	2020	FRONTIERS IN PSYCHOLOGY	10.3389/fpsyg.2020.01168
**19**	2020	EUROPEAN RADIOLOGY	10.1007/s00330-020-07033-y
**20**	2020	JOURNAL OF HUMAN GENETICS	10.1038/s10038-020-0808-9
**21**	2020	NUTRIENTS	10.3390/nu12072016
**22**	2020	DENTAL AND MEDICAL PROBLEMS	10.17219/dmp/119743
**23**	2020	EUROPEAN HEART JOURNAL	10.1093/eurheartj/ehaa508
**24**	2020	JOURNAL OF THROMBOSIS AND THROMBOLYSIS	10.1007/s11239-020-02171-y
**25**	2020	BRAIN BEHAVIOR AND IMMUNITY	10.1016/j.bbi.2020.05.038
26	*2020*	JOURNAL OF INFECTION AND PUBLIC HEALTH	10.1016/j.jiph.2020.06.021
**27**	2020	JOURNAL OF PUBLIC HEALTH	10.1093/pubmed/fdaa070
**28**	2020	JOURNAL OF COMMUNITY HEALTH	10.1007/s10900-020-00881-1
**29**	2020	RESPIRATORY RESEARCH	10.1186/s12931-020-01429-6
**30**	2020	JOURNAL OF MICROBIOLOGY IMMUNOLOGY AND INFECTION	10.1016/j.jmii.2020.03.026
**31**	2021	JOURNAL OF ENDOCRINOLOGICAL INVESTIGATION	10.1007/s40618-020-01370-x
**32**	2020	JOURNAL OF INTENSIVE CARE	10.1186/s40560-020-00466-z
**33**	2020	AMERICAN JOURNAL OF ROENTGENOLOGY	10.2214/AJR.20.23078
**34**	2020	RADIOLOGIA MEDICA	10.1007/s11547-020-01232-9
**35**	2020	INTERNATIONAL JOURNAL OF INFECTIOUS DISEASES	10.1016/j.ijid.2020.05.076
36	*2020*	QUANTITATIVE IMAGING IN MEDICINE AND SURGERY	10.21037/qims-20-564
**37**	2020	PLOS ONE	10.1371/journal.pone.0239252
**38**	2020	INTERNATIONAL JOURNAL OF MEDICAL SCIENCES	10.7150/ijms.46614
**39**	2020	CUREUS	10.7759/cureus.7923
**40**	2021	THYROID	10.1089/thy.2020.0363
**41**	2020	LEUKEMIA	10.1038/s41375-020-0911-0
**42**	2020	BMC INFECTIOUS DISEASES	10.1186/s12879-020-05128-x
**43**	2020	BIOMED RESEARCH INTERNATIONAL	10.1155/2020/6159720
**44**	2020	JOURNAL OF PHARMACEUTICAL ANALYSIS	10.1016/j.jpha.2020.03.004
**45**	2020	RESPIRATION	10.1159/000509223
46	*2020*	INFECTIOUS DISEASES AND THERAPY	10.1007/s40121-020-00324-3
**47**	2020	ENVIRONMENT DEVELOPMENT AND SUSTAINABILITY	10.1007/s10668-020-00878-9
**48**	2020	RESPIRATORY RESEARCH	10.1186/s12931-020-01428-7
**49**	2020	JOURNAL OF PERINATAL MEDICINE	10.1515/jpm-2020-0182
**50**	2020	NUTRIENTS	10.3390/nu12072098
**51**	2020	AEROSOL AND AIR QUALITY RESEARCH	10.4209/aaqr.2020.05.0218
**52**	2020	GEOPHYSICAL RESEARCH LETTERS	10.1029/2020GL088533
**53**	2020	EUROPEAN JOURNAL OF ENDOCRINOLOGY	10.1530/EJE-20-0335
**54**	2020	ELECTRONIC JOURNAL OF GENERAL MEDICINE	10.29333/ejgm/8223
**55**	2020	BIOSCIENCE TRENDS	10.5582/bst.2020.03086
**56**	2020	THERANOSTICS	10.7150/thno.46569
**57**	2021	SLEEP MEDICINE	10.1016/j.sleep.2020.05.018
**58**	2020	EUROPEAN JOURNAL OF HEART FAILURE	10.1002/ejhf.1990
**59**	2020	CLINICA CHIMICA ACTA	10.1016/j.cca.2020.06.012
**60**	2020	AMERICAN JOURNAL OF OTOLARYNGOLOGY	10.1016/j.amjoto.2020.102612
**61**	2020	AGING-US	
**62**	2020	ANTIMICROBIAL AGENTS AND CHEMOTHERAPY	10.1128/AAC.01177-20
**63**	2020	JOURNAL OF OTOLARYNGOLOGY-HEAD & NECK SURGERY	10.1186/s40463-020-00449-y
**64**	2020	ENVIRONMENT DEVELOPMENT AND SUSTAINABILITY	10.1007/s10668-020-00849-0
**65**	2020	BMC PUBLIC HEALTH	10.1186/s12889-020-09392-z
**66**	2020	ANNALS OF INTENSIVE CARE	10.1186/s13613-020-00716-1
**67**	2020	INTERNATIONAL JOURNAL OF MENTAL HEALTH AND ADDICTION	10.1007/s11469-020-00355-4
**68**	2020	EUROPEAN REVIEW FOR MEDICAL AND PHARMACOLOGICAL SCIENCES	10.26355/eurrev_202009_22874

**69**	2020	JOURNAL OF AFFECTIVE DISORDERS	10.1016/j.jad.2020.06.047
**70**	2020	JOURNAL OF PSYCHIATRIC RESEARCH	10.1016/j.jpsychires.2020.06.022
**71**	2020	INTENSIVE CARE MEDICINE	10.1007/s00134-020-06229-6
**72**	2020	ACS CHEMICAL NEUROSCIENCE	10.1021/acschemneuro.0c00447
**73**	2020	VACCINES	10.3390/vaccines8030378
**74**	2020	INFECTIOUS DISEASES OF POVERTY	10.1186/s40249-020-00703-5
75	*2020*	ERJ OPEN RESEARCH	10.1183/23120541.00260-2020
**76**	2020	FRONTIERS IN PSYCHIATRY	10.3389/fpsyt.2020.00751
**77**	2020	CLINICA CHIMICA ACTA	10.1016/j.cca.2020.04.020
**78**	2020	EXPERIMENTAL HEMATOLOGY & ONCOLOGY	10.1186/s40164-020-00172-4
**79**	2020	EUROPEAN RADIOLOGY	10.1007/s00330-020-07013-2
**80**	2020	EMERGING MICROBES & INFECTIONS	10.1080/22221751.2020.1771219
**81**	2020	SHOCK	10.1097/SHK.0000000000001562
**82**	2020	JOURNAL OF AFFECTIVE DISORDERS	10.1016/j.jad.2020.06.035
**83**	2020	JOURNAL OF INFECTION AND PUBLIC HEALTH	10.1016/j.jiph.2020.05.029
**84**	2020	INFECTIOUS DISEASES OF POVERTY	10.1186/s40249-020-00723-1
**85**	2020	INTERNATIONAL JOURNAL OF MENTAL HEALTH AND ADDICTION	10.1007/s11469-020-00371-4
**86**	2020	RESPIRATORY RESEARCH	10.1186/s12931-020-01427-8
**87**	2020	WESTERN JOURNAL OF EMERGENCY MEDICINE	10.5811/westjem.2020.5.47780
**88**	2020	JOURNAL OF CLINICAL MEDICINE	10.3390/jcm9061781
**89**	2021	JOURNAL OF AUTOIMMUNITY	10.1016/j.jaut.2020.102560
**90**	2020	INTERNATIONAL JOURNAL OF INFECTIOUS DISEASES	10.1016/j.ijid.2020.09.014
**91**	2020	PROCEEDINGS OF THE NATIONAL ACADEMY OF SCIENCES OF THE UNITED STATES OF AMERICA	10.1073/pnas.2010540117
**92**	2020	JOURNAL OF INFECTION	10.1016/j.jinf.2020.08.013
**93**	2020	CLINICAL MEDICINE	10.7861/clinmed.2020-0346
**94**	2020	JOURNAL OF CLINICAL VIROLOGY	10.1016/j.jcv.2020.104542
**95**	2020	VACCINE	10.1016/j.vaccine.2020.06.069
**96**	2020	INTERNATIONAL JOURNAL OF ENVIRONMENTAL RESEARCH AND PUBLIC HEALTH	10.3390/ijerph17145170
**97**	2020	WORLD JOURNAL OF EMERGENCY SURGERY	10.1186/s13017-020-00323-2
**98**	2020	JOURNAL OF TRANSLATIONAL MEDICINE	10.1186/s12967-020-02418-5
**99**	2020	INTERNATIONAL JOURNAL OF ENVIRONMENTAL RESEARCH AND PUBLIC HEALTH	10.3390/ijerph17113903
**100**	2020	MEDICAL SCIENCE MONITOR	10.12659/MSM.925669
**101**	2020	CLINICA CHIMICA ACTA	10.1016/j.cca.2020.07.002
**102**	2020	CLINICA CHIMICA ACTA	10.1016/j.cca.2020.08.008
**103**	2020	PEERJ	10.7717/peerj.10038
**104**	2021	JOURNAL OF MEDICAL VIROLOGY	10.1002/jmv.26365
**105**	2020	BMC ORAL HEALTH	10.1186/s12903-020-01187-3
